# Synthesis and characterization of integrated layered nanocomposites for lithium ion batteries

**DOI:** 10.1186/1556-276X-7-60

**Published:** 2012-01-05

**Authors:** Jihyeon Gim, Jinju Song, Hyosun Park, Jungwon Kang, Kangkun Kim, Vinod Mathew, Jaekook Kim

**Affiliations:** 1 Department of Materials Science and Engineering, Chonnam National University, 300 Yongbongdong, Bukgu, Gwangju, 500-757, South Korea

**Keywords:** lithium ion batteries, cathodes, nanocomposites, coprecipitation

## Abstract

The series of Li[Ni*_x_*M*_x_*Li_1/3-*x*_Mn_2/3-*x*_]O_2 _cathodes, where M is cobalt or chromium with a wide compositional range *x *from 0 to 0.33, were prepared by hydroxide coprecipitation method with subsequent quenching. The sample structures were investigated using X-ray diffraction results which were indexed completely on the basis of a trigonal structure of space group $\bar{\text{R3m}}$ with monoclinic C2/m phase as expected. The morphologies and electrochemical properties of the samples obtained were compared as the value of *x *and substituted transition metal. The particle sizes of cobalt-substituted Li[Ni*_x_*Co*_x_*Li_1/3-*x*_Mn_2/3-*x*_]O_2 _samples are much smaller than those of the Li[Ni*_x_*Cr*_x_*Li_1/3-*x*_Mn_2/3-*x*_]O_2 _system. The electrode containing Li[Ni*_x_*Co*_x_*Li_1/3-*x*_Mn_2/3-*x*_]O_2 _with *x *= 0.10 delivered a discharge capacity of above 200 mAh/g after 10 cycles due to the activation of Li_2_MnO_3_.

**PACS: **82.47.Aa; 82.47.-a; 82.45.Fk.

## Introduction

The development of rechargeable lithium ion batteries depends critically on the technological advances in electrode materials. Over the years, several compounds such as spinel LiMn_2_O_4_, olivine LiFePO_4 _[1], and layered LiCoO_2 _and LiNiO_2 _have been studied extensively by many researchers as cathode materials for lithium ion batteries. In fact, LiMn_2_O_4 _and LiFePO_4 _have distinct advantages of being cost-effective and environmentally benign. However, LiMn_2_O_4 _suffers from capacity fading due to the dissolution of manganese and Jahn-Teller distortion [2,3], while LiFePO_4 _delivers insufficient capacity and low electronic conductivity [4].

Commercially used LiCoO_2 _cathode has advantages of easy synthesis and excellent lithium ion mobility though challenging issues of stability, achieving practical capacities, and environmental risks need to be addressed [2]. The layer-structured rhombohedral LiMnO_2 _($\bar{\text{R3m}}$) attracts interest as a potential cathode due to its cost effectiveness and relatively high capacity, but it exhibits severe capacity fading during extended cycling. More precisely, its discharge behavior during electrochemical cycling needs significant improvement. The strategies to overcome such limitations in rhombohedral LiMnO_2 _have been focused on metal ion substitution [5,6]. Due to its higher theoretical capacity, LiNiO_2 _has also been investigated as an alternative cathode to commercial LiCoO_2_. However, it is complicated to synthesize a pure-layered structure with a well-ordered phase because of severe cationic disordering between nickel and lithium ions that occurs due to the ionic radii values of Ni^2+^(0.069 nm) and Li^+^(0.068 nm) being almost similar. Further, capacity fading occurs during discharge since the electronic state in low spin Ni^3+ ^serves as the satisfactory condition for the Jahn-Teller distortion observed in the spinel LiMn_2_O_4_.

In light of the above discussions, many researchers have investigated on the strategies to replace LiCoO_2_. First, alien transition metal ions such as Ni, Mn, and Cr could be introduced in order to exploit their advantages of stable and high redox-couple properties. Second, by combining stable Li_2_MnO_3 _as an inactive frame with layered LiMO_2_, lithium-saturated solid solutions or nanocomposite *x*Li_2_MnO_3_·(1-*x*)LiMO_2 _with prolonged structural integrities have been researched to take advantage of their stable and rigid structure [7-11]. Here, Li_2_MnO_3_, which has a layered rock salt structure (space group $\bar{\text{R3m}}$) with a monoclinic phase (C2/m), can be represented in layered form as Li[Li_1/3_Mn_2/3_]O_2_. Further, the nanocomposites can be represented by the notation, Li[M_1-*x*_Li_*x*/3 _Mn_2*x*/3_]O_2 _with a layered structure [12-14]. Our earlier work was focused on investigating one such nanocomposite electrode namely, 0.4Li_2_MnO_3_·0.6LiMO_2 _(M = Ni_1/3_Co_1/3_Mn_1/3 _and Ni_1/3_Cr_1/3_Mn_1/3_) [13]. The encouraging results obtained from that study led us to investigate the physicochemical properties of the doped nanocomposites with a layered structure over a range of stoichiometric compositions.

Therefore, the present work reports on the synthesis and systematic investigations on the structure, morphology, and electrochemical performances of an integrated layered nanocomposite system, *viz *Li[Ni*_x_*M*_x_*Li_1/3-*x*_Mn_2/3-*x*_]O_2_, where M is cobalt or chromium with a wide compositional range *x *from 0 to 0.33. Ultimately, it is aimed to arrive at the optimized compositions (*x*) of Co and Cr in the integrated nanocomposite that exhibit impressive electrochemical properties.

## Methods

### Synthesis

Lithium hydroxide monohydrate (98.0% to approximately 102.0%; Junsei Chemical Co., Ltd., Chuo-ku, Tokyo, Japan), manganese acetate tetrahydrate (97%; Yakuri Pure Chemicals Co., Ltd., Kyoto, Japan), nickel acetate tetrahydrate (98.0%, Junsei Chemical Co., Ltd.), Cobalt acetate tetrahydrate (98.5%, Junsei Chemical Co., Ltd.) and Chromium acetate (22% as Cr, Wako Pure Chemical Industries, Ltd., Chuo-ku, Osaka, Japan) were used as precursors for the solution synthetic method. The samples with different stoichiometric compositions in the layered Li[Ni*_x_*M*_x_*Li_1/3-*x*_Mn_2/3-*x*_]O_2 _system where *x *= 0, 0.05, 0.1, 0.17, 0.24, and 0.33 were prepared by coprecipitation method. In brief, the transition metal acetate precursors and lithium hydroxide were dissolved separately in distilled water. The aqueous solution of lithium hydroxide was then slowly dripped into the transition metal solution to facilitate hydroxide coprecipitation at room temperature for 24 h. The precipitated solution was subsequently dried in an oven at 85°C to evaporate residual water, and the dried powders were ground well before heating at 600°C for 3 h to eliminate undesired organic materials that remained. The heated powders were ground completely and then fired at 900°C for 12 h for crystallization. The resultant powders were obtained after quenching the fired powders using two copper plates in air and subsequent grinding. The final products were obtained after washing with distilled water to remove unwanted impurities such as Li_2_CrO_4 _and subsequent vacuum drying at 120°C.

### Structural and physical characterization

The crystalline nature of the obtained samples in the Li[Ni*_x_*M*_x_*Li_1/3-*x*_Mn_2/3-*x*_]O_2 _system were characterized by X-ray diffraction [XRD] using a Shimadzu X-ray diffractometer (Shimadzu Corporation, Nakagyo-ku, Kyoto, Japan) with Ni-filtered Cu-K*α *radiation (*λ *= 1.5406 Å) operating at 40 kV and 30 mA within the scanning range angle from 10° to 80° (2*θ*). Inductively coupled plasma atomic emission spectrometer [ICP-AES] analysis utilizing PerkinElmer OPTIMA 4300 DV (PerkinElmer, Waltham, MA, USA) was performed to confirm the compositions of the obtained materials. The particle morphologies and sizes were observed by field-emission scanning electron microscopy [FE-SEM] using the HITACHI S-4700 instrument (Hitachi High-Tech, Minato-ku, Tokyo, Japan). The sample surface areas were measured by the Brunauer Emmett and Teller [BET] method using a surface area analyzer (ASAP 2020, Micromeritics Instrument Co., Norcross, GA, USA).

### Electrochemical characterization

The electrochemical properties of the cathodes fabricated from the samples in the Li[Ni*_x_*M*_x_*Li_1/3-*x*_Mn_2/3-*x*_]O_2 _system were evaluated using the NAGANO battery tester system 2004H equipment (NAGANO KEIKI Co., LTD, Ohta-ku, Tokyo, Japan). The fabricated cathode consisted of 72 wt.% active materials, 10 wt.% conductive carbon (Ketjen black), and 18 wt.% polytetrafluoroethylene as binder. The pasted film was then pressed onto a stainless steel mesh with a 2-cm^2 ^area and dried under vacuum at 120°C for 12 h. The electrolyte employed was a 1:1 (*v*/*v*) mixture of ethylene carbonate and dimethyl carbonate containing 1 M LiPF_6_. A 2032 coin-type cell which consists of the cathode and lithium metal anode separated by a polymer membrane was fabricated in an Ar-filled glove box and aged for 12 h. The cells assembled were tested with 0.1 mA/cm^2 ^of current density in the voltage range from 2.0 to 4.8 V.

## Results and discussion

### The Li[Ni*_x_*Co*_x_*Li_1/3-*x*_Mn_2/3-*x*_]O_2 _system

**Figure 1:**
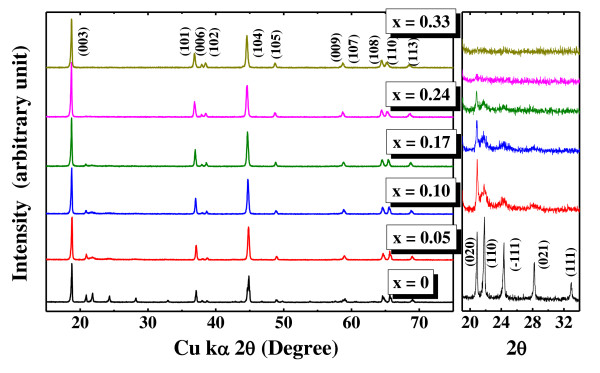
**XRD patterns of Li[Ni*_x_*Co*_x_*Li_1/3-*x*_Mn_2/3-*x*_]O_2 _system synthesized by coprecipitation and magnified image in the 19° to 34°(2*θ*) region**.

Figure 1 shows the XRD patterns of layered nanocomposite powders obtained by coprecipitation and belonging to the Li[Ni*_x_*Co*_x_*Li_1/3-*x*_Mn_2/3-*x*_]O_2 _system. All diffraction peaks of the prepared samples were assigned to the expected reflections of trigonal ($\bar{\text{R3m}}$) and monoclinic (C2/m) phases simultaneously, except for the sample with composition *x *= 0. Particularly, a magnified view of the scanning angles ranging from 2*θ *= 19° to 34° indicate peaks arising due to the super-lattice ordering of Li^+ ^and Mn^4+ ^occurring in the transition metal layers. Li_2_MnO_3 _can be represented as Li[Li_1/3_Mn_2/3_]O_2_, a layered phase possessing long-range ordering in the transition metal layers. Such a cation ordering can correspond to well-resolved characteristic peaks at specific angles in the XRD patterns. These peaks indicating long-range ordering are distinctly visible and sharper in pure Li_2_MnO_3 _(*x *= 0), when compared with the other samples in this system. As the XRD patterns of the samples with increasing concentrations of *x *are viewed progressively, the characteristic peaks corresponding to cation ordering undergo a significant variation in their intensities. In fact, the peaks which indicate the cation ordering in transition metal layer disappear gradually, as we observe the characteristic peaks of the samples with increasing concentrations of *x *in the magnified image. To confirm the stoichiometric composition of the synthesized materials, ICP-AES analysis was performed, and the results are summarized in Table 1. The ICP results revealed that the observed stoichiometric composition for transition metals in all the samples matched well with the calculated values. Despite the fact that an excess 3 wt.% lithium precursor was used as starting material, the experimental lithium content in all samples was slightly lower than the corresponding theoretical values. This lower lithium content most probably resulted from the evaporation loss of lithium during heat treatment at elevated temperatures. The possibilities for such lithium losses during high temperature synthesis of layered electrodes have been reported [12,15].

**Table 1:** The ICP data confirming the stoichiometries of the prepared Co-doped samples and the corresponding BET values.

		Measured stoichiometry (Ref:Mn)				a_s_, BET (m^2^/g)
			
Sample	Target stoichiometry	Li	Ni	Co	Mn	
*x *= 0.33	Li[Ni_0.33_Co_0.33_Mn_0.33_]O_2_	0.85	0.34	0.35	0.33	2.87
*x *= 0.24	Li[Ni_0.24_Co_0.24_Li_0.09_Mn_0.42_]O_2_	0.90	0.24	0.25	0.42	2.95
*x *= 0.17	Li[Ni_0.17_Co_0.17_Li_0.17_Mn_0.50_]O_2_	0.96	0.16	0.17	0.50	2.53
*x *= 0.10	Li[Ni_0.10_Co_0.10_Li_0.23_Mn_0.56_]O_2_	1.05	0.10	0.10	0.56	2.33
*x *= 0.05	Li[Ni_0.05_Co_0.05_Li_0.29_Mn_0.62_]O_2_	1.13	0.04	0.05	0.62	1.81
*x *= 0	Li[Li_0.33_Mn_0.67_]O_2_	1.20	0	0	0.67	0.29

**Figure 2:**
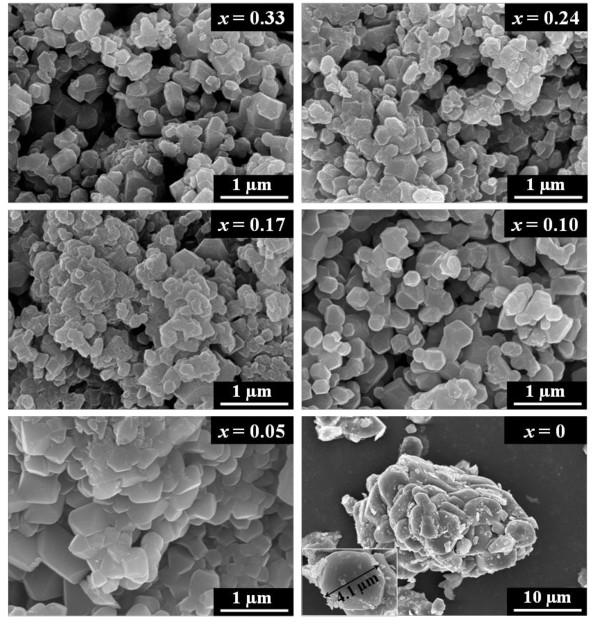
**FE-SEM images of Li[Ni*_x_*Co*_x_*Li_1/3-*x*_Mn_2/3-*x*_]O_2 _system synthesized by coprecipitation**.

The morphology and size distribution of the Li[Ni*_x_*Co*_x_*Li_1/3-*x*-_Mn_2/3-*x*_]O_2 _system were examined by FE-SEM and is shown in Figure 2. From the SEM results, it is observed that the average particle size of the parent Li_2_MnO_3 _(sample with *x *= 0) is in the range of 4 μm. On doping with Co , the particle sizes of the doped samples tend to decrease, which might probably be due to the comparatively smaller ionic radius of Co^3+ ^(0.053 nm) than that of Ni^2+ ^(0.07 nm). A similar trend observed by researchers has been reported for Cr-doping in layered lithium manganese oxides [16,17]. The surface areas pertaining to the prepared samples which were calculated using the BET method indicate that the obtained values for the doped samples exceed those of the parent sample by an order of magnitude, as evidenced from Table 1. This trend clearly further indicates that Co-doped samples possess smaller particle sizes than the undoped sample. However, among the doped samples, the surface area values undergo a marginal increase in the same order of magnitude for higher dopant concentrations until the value experiences a slight decline for the highest doping concentration of *x *= 0.33. Nevertheless, further investigations are required to understand the correlation between particle size and concentration of Co dopant. As observed from the well-developed crystal facets of the particles, the tetrakai-dodecahedral morphology is confirmed in the doped samples. The particle sizes are observed to roughly vary between 200 and 500 nm. The absence of a noticeable variation in the obtained morphologies of the doped samples indicates that varying the concentration of Co doping hardly introduces significant changes in the particle morphologies.

**Figure 3:**
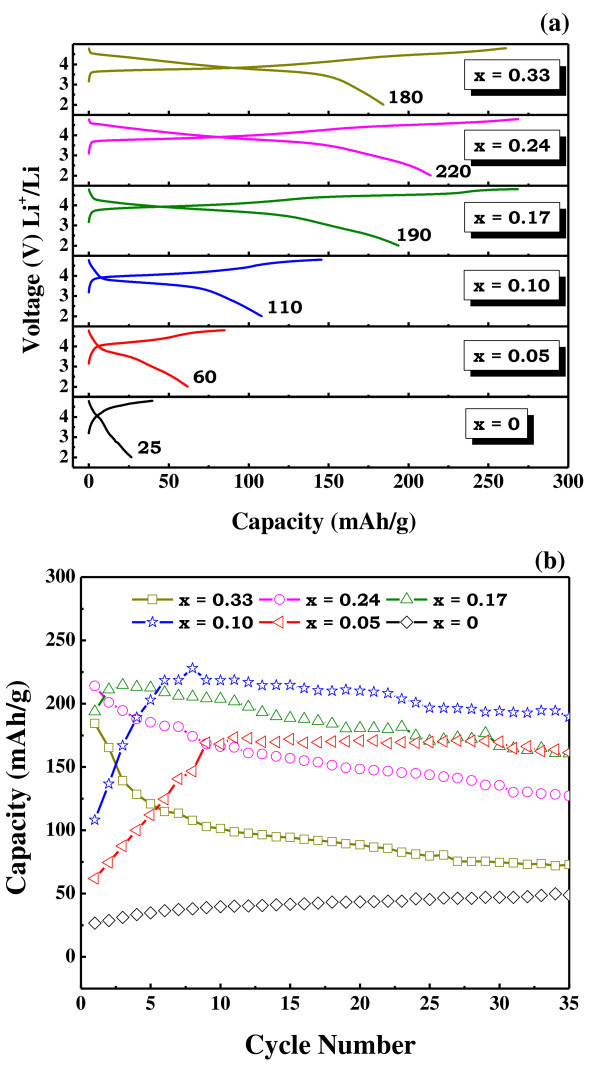
**Electrochemical properties of Li[Ni*_x_*Co*_x_*Li_1/3-*x*_Mn_2/3-*x*_]O_2 _system with initial charge and discharge profiles (a) and cycleabilities (b)**.

The initial charge/discharge profiles for all the prepared electrodes in the Li[Ni*_x_*Co*_x_*Li_1/3-*x*_Mn_2/3-*x*_]O_2 _system and their cycleabilities are shown in Figure 3. The charge capacities tend to increase until the intermediate concentrations of Co and the values tend to reach saturation for higher Co contents. However, a different trend follows for the obtained discharge capacities. As the Co content (*x*) in the nanocomposite increased, a distinct improvement in the discharge capacities was observed until *x *= 0.24; beyond which, a drop in the discharge capacity occurred. Hence, the coulombic efficiencies in the doped samples were apparently higher (> 70%) than those observed in the pure sample (66%) which suggests that the higher efficiencies are probably associated with Co doping. The smaller the particle size, the higher the electrode/electrolyte interfacial areas; hence, shorter are the Li-ion diffusion paths. The reduced ion migration pathways lead to effective ion diffusion and ultimately enhance material properties/performances. However, the significant initial irreversible capacities observed in the voltage profiles of such layered nanocomposites arise mainly from the oxygen loss occurring at extended charge cycling (> 4.5 V) [12,14]. A maximum initial discharge and charge capacities of 270 and 220 mAh/g were registered for the sample with the composition *x *= 0.24. The specific capacity drop beyond this particular composition is most probably associated with the particular compositional ratio of the nanocomposite. In fact, beyond this composition, the Li_2_MnO_3 _content decreases, as seen from the XRD result. The electrochemically inactive Li_2_MnO_3 _in conjunction with the appropriate LiMO_2 _composition enhances the electrochemical properties of the final nanocomposite though other factors such as particle size and distribution need to be considered. Although the highest charge and discharge capacities were observed for the sample with the composition *x *= 0.24, the values steadily declined after few initial cycles. However, the capacities of the sample with low Co content (*x *= 0.05 and 0.10) increased gradually and steadied under subsequent cycling. On cycling the electrodes for 35 cycles, the capacities maintained by the latter samples were far better than those of the former. For instance, the capacity of the sample with high Co content underwent a decline from the initial value of 214 mAh/g to a final value of 127.12 mAh/g after the first 35 cycles. In contrast, the sample with a lower concentration of Co (*x *= 0.10), which delivered an initial capacity of 108.12 mAh/g, registered a higher capacity of 189.46 mAh/g after 35 cycles, the value achieved being 49% higher than that attained by the former under the same electrochemical conditions. The gradual rise in the capacities in the sample with low Co content has been attributed to the activation of these electrodes on repeated cycling. These results led us to conclude that the sample with Co content (*x*) varying between 0.05 and 0.10 in the Li[Ni*_x_*Co*_x_*Li_1/3-*x*_Mn_2/3-*x*_]O_2 _system displayed an optimized electrochemical performance compared to the other counterparts.

### The Li[Ni*_x_*Cr*_x_*Li_1/3-*x*_Mn_2/3-*x*_]O_2 _system

**Figure 4:**
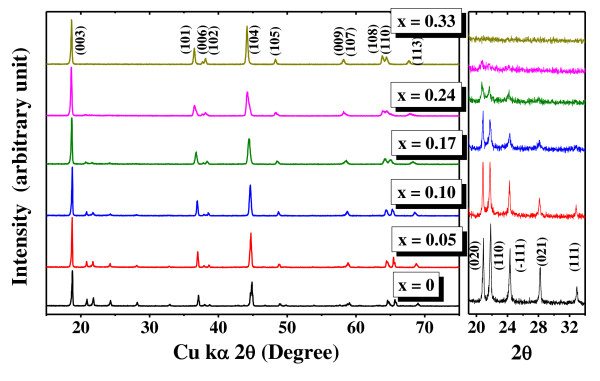
**XRD patterns of Li[Ni*_x_*Cr*_x_*Li_1/3-*x*_Mn_2/3-*x*_]O_2 _system synthesized by coprecipitation and magnified image in the 19° to 34°(2*θ*) region**.

**Figure 5:**
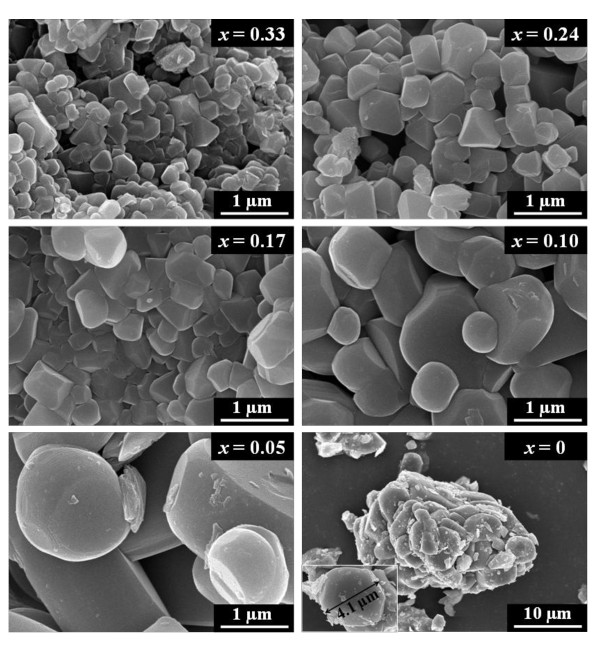
**FE-SEM images of Li[Ni*_x_*Cr*_x_*Li_1/3-*x*_Mn_2/3-*x*_]O_2 _system synthesized by coprecipitation**.

**Figure 6:**
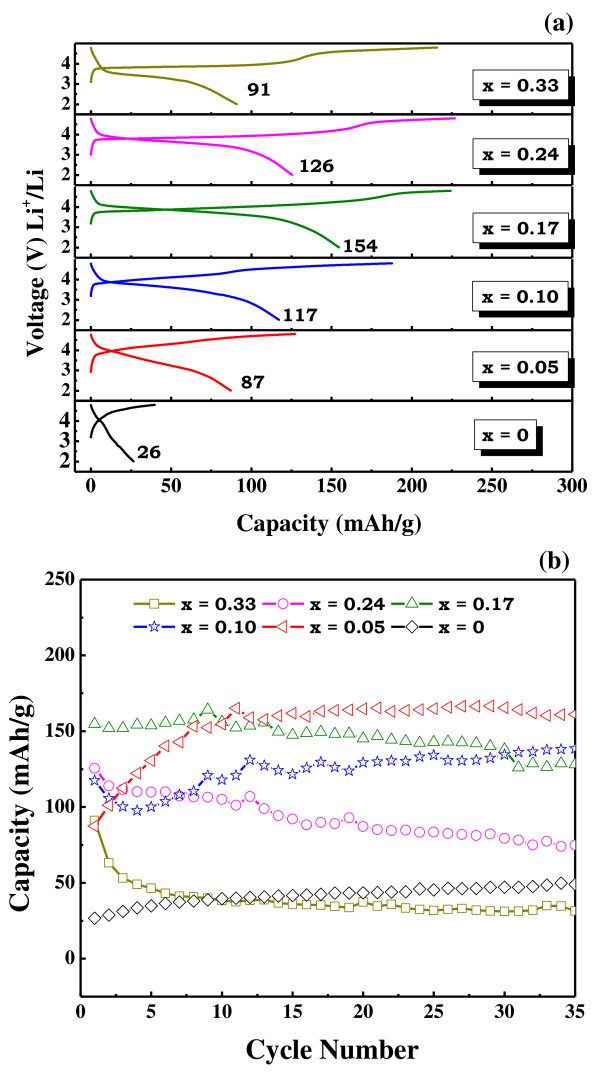
**Electrochemical properties of Li[Ni*_x_*Cr*_x_*Li_1/3-*x*_Mn_2/3-*x*_]O_2 _system with initial charge and discharge profiles (a) and cycleabilities (b)**.

The XRD profiles, ICP-AES results, SEM images, and electrochemical properties of Li[Ni*_x_*Cr*_x_*Li_1/3-*x*_Mn_2/3-*x*_]O_2 _system, where *x *= 0, 0.05, 0.1, 0.17, 0.24, and 0.33, were obtained to compare with the results obtained for the cobalt-containing nanocomposite system, *viz *Li[Ni*_x_*Co*_x_*Li_1/3-*x*_Mn_2/3-*x*_]O_2_. The XRD patterns of the Cr-doped samples, depicted in Figure 4, follow a similar trend to those observed in the Co-doped system; hence, the explanation of the XRD results holds valid for the Cr-doped system as in the case of the former system. The obtained ICP data, summarized in Table 2, confirm the stoichiometries, excepting the evaporation losses in the case of lithium. The FE-SEM images of the Cr-doped nanocomposites are shown in Figure 5. It appears that the doping of Cr leads to a slight reduction in the particle size, and the BET surface area values in Table 2 tend to confirm the observation. However, on comparison of the SEM images of the Co-doped and Cr-doped layered composites in Figures 2 and 5, respectively, it is observed that the particle sizes of the Li[Ni*_x_*Cr*_x_*Li_1/3-*x*_Mn_2/3-*x*_]O_2 _system are larger, with diameters of 300 nm to 1 μm, than those of the Li[Ni*_x_*Co*_x_*Li_1/3-*x*_Mn_2/3-*x*_]O_2 _system. This might probably be due to the apparently smaller ionic radius of Co^3+ ^(0.053 nm) than of Cr^3+ ^(0.061 nm), and this observation is in congruence with our earlier report on Co/Cr-doped layered nanocomposites [13]. The electrochemical properties in Figure 6 of the Cr-substituted nanocomposite system exhibited apparently lower performances compared with those in the Co-contained nanocomposite system. In the Li[Ni*_x_*Cr*_x_*Li_1/3-*x*_Mn_2/3-*x*_]O_2 _system, the highest initial discharge capacity of 155 mAh/g was observed for the sample with the Cr composition *x *= 0.17. However, on completion of the initial 35 cycles, a capacity retention of 71% was observed (120 mAh/g). Whereas the sample with a low Cr content (*x *= 0.05), which delivered a lower initial discharge capacity of 87.46 mAh/g, registered higher capacities for 10 consecutive cycles and stabilized thereafter at 150 mAh/g, the value being 33% much higher than that observed under similar electrochemical conditions for the sample with the highest initial discharge capacity (*x *= 0.17). This behavior is similar to the case observed for the samples in the Co-doped nanocomposite system. Further, the enhanced electrochemical abilities of the Co-doped system may probably be due to the smaller particle sizes achieved by the coprecipitation process.

**Table 2:** The ICP data confirming the stoichiometries of the prepared Cr-doped samples and the corresponding BET values.

		Measured stoichiometry (Ref:Mn)				a_s_, BET (m^2^/g)
			
Sample	Target stoichiometry	Li	Ni	Cr	Mn	
*x *= 0.33	Li[Ni_0.33_Cr_0.33_Mn_0.33_]O_2_	0.87	0.34	0.35	0.33	0.96
*x *= 0.24	Li[Ni_0.24_Cr_0.24_Li_0.09_Mn_0.42_]O_2_	0.93	0.24	0.25	0.42	1.44
*x *= 0.17	Li[Ni_0.17_Cr_0.17_Li_0.17_Mn_0.50_]O_2_	1.01	0.16	0.17	0.50	1.64
*x *= 0.10	Li[Ni_0.10_Cr_0.10_Li_0.23_Mn_0.56_]O_2_	1.04	0.10	0.10	0.56	0.73
*x *= 0.05	Li[Ni_0.05_Cr_0.05_Li_0.29_Mn_0.62_]O_2_	1.09	0.04	0.05	0.62	0.73
*x *= 0	Li[Li_0.33_Mn_0.67_]O_2_	1.20	0	0	0.67	0.29

## Conclusions

In summary, structurally integrated nanocomposite materials belonging to the system, Li[Ni*_x_*M*_x_*Li_1/3-*x*_Mn_2/3-*x*_]O_2 _where M is Co or Cr, were synthesized by hydroxide coprecipitation method and subsequent quenching process. The XRD patterns of all the prepared nanocomposite samples were well indexed to the trigonal (R3m) structure and monoclinic (C2/m) phase. However, obtaining the target stoichiometric composition is not trivial due to the reactivity of lithium at elevated temperatures. The average particle size of the crystallites in the Li[Ni*_x_*M*_x_*Li_1/3-*x*_Mn_2/3-*x*_]O_2 _system is dependent on whether the transition metal of M is Co or Cr. In the case of the Co-substituted system, particle sizes were much smaller than those in the Li[Ni*_x_*Cr*_x_*Li_1/3-*x*_Mn_2/3-*x*_]O_2 _system. Consequently, impressive electrochemical properties were attained since discharge capacities as high as 200 mAh/g and above were registered after the initial 10 cycles for the sample with *x *= 0.10 in the Li[Ni*_x_*Co*_x_*Li_1/3-*x*_Mn_2/3-*x*_]O_2 _system. Further studies focused not only on the co-existence of R3m and C2/m, but also investigation on the local structure characterization will be required in detail using advanced analysis such as transmission electron microscopy and nuclear magnetic resonance.

## Competing interests

The authors declare that they have no competing interests.

## Authors' contributions

JKK directed the research. JG analyzed the results and wrote the paper. JS, HP, JWK, and KK participated in the characterization of samples and carried out experiments. VM contributed to the technical discussions. All the authors have read and approved the final manuscript.
